# Altered functional differentiation of mesoangioblasts in a genetic myopathy

**DOI:** 10.1111/jcmm.12023

**Published:** 2013-02-07

**Authors:** Claudia Altomare, Lucio Barile, Marcella Rocchetti, Luca Sala, Stefania Crippa, Maurilio Sampaolesi, Antonio Zaza

**Affiliations:** a Department of Biotechnologies and Biosciences, University of Milano-BicoccaMilan, Italy; b Molecular Cardiology Laboratory, Fondazione Cardiocentro TicinoLugano, Switzerland; c Translational Cardiomyology, Stem Cell Research Institute, Catholic University of LeuvenLeuven, Belgium

**Keywords:** cardiac mesoangioblasts, differentiation switch, β-sarcoglycan

## Abstract

Mutations underlying genetic cardiomyopathies might affect differentiation commitment of resident progenitor cells. Cardiac mesoangioblasts (cMabs) are multipotent progenitor cells resident in the myocardium. A switch from cardiac to skeletal muscle differentiation has been recently described in cMabs from β-sarcoglycan-null mice (βSG^−/−^), a murine model of genetic myopathy with early myocardial involvement. Although complementation with βSG gene was inconsequential, knock-in of miRNA669a (missing in βSG^−/−^ cMabs) partially rescued the mutation-induced molecular phenotype. Here, we undertook a detailed evaluation of functional differentiation of βSG^−/−^ cMabs and tested the effects of miRNA669a-induced rescue *in vitro*. To this end, cMabs were compared with neonatal cardiomyocytes (CMs) and skeletal muscle C2C12 cells, representative of cardiac and skeletal muscle respectively. Consistent with previous data on molecular patterns, electrophysiological and Ca^2+^-handling properties of βSG^−/−^ cMabs were closer to C2C12 cells than to CM ones. Nevertheless, subtler aspects, including action potential contour, Ca^2+^-spark properties and RyR isoform expression, distinguished βSG^−/−^ cMabs from C2C12 cells. Contrary to previous reports, wild-type cMabs failed to show functional differentiation towards either cell type. Knock-in of miRNA669a in βSG^−/−^ cMabs rescued the wild-type functional phenotype, *i.e*. it completely prevented development of skeletal muscle functional responses. We conclude that miRNA669a expression, ablated by βSG deletion, may prevent functional differentiation of cMabs towards the skeletal muscle phenotype.

## Introduction

In spite of the low intrinsic regenerative potential of cardiac muscle, undifferentiated self-renewing cells, capable of multi-lineage differentiation, can be found within the adult myocardium. The possibility that such ‘Cardiac Progenitor Cells’ (CPCs) may be particularly prone to differentiate into cardiomyocytes has received considerable attention [1–4]. Vessel-associated progenitor cells, named ‘mesoangioblasts’ (Mabs) [5], have been isolated as a resident population from adult skeletal [6, 7] and cardiac muscle [8]. Cardiac-resident Mabs (cMabs) are CPCs with a specific markers profile and, despite their association with vessels, they are reported to spontaneously acquire cardiomyocyte molecular and functional phenotypes when differentiated *in vitro* [8, 9]. On the other hand, skeletal mesoangioblasts (skMabs) differentiated into skeletal myotubes when co-cultured with a skeletal cell line (C2C12), or transfected with skeletal transcriptional regulator (MyoD) [7]. These observations support the intriguing hypothesis of a role of tissue-specific factors in committing resident multipotent cells to their differentiation fate.

β-sarcoglycan (βSG) is a protein of the dystrophin complex with still undefined functions [10]. βSG-null mouse (βSG^−/−^) develops a skeletal myopathy with early cardiac involvement [11]. We recently found that cMabs, isolated from the atria and ventricles of βSG^−/−^ mice spontaneously differentiate into skeletal myotubes [12], instead of cardiomyocytes. This surprising observation suggested at first that βSG itself might act as a differentiation switch. However, further analysis showed that βSG^−/−^ cMabs had deficient expression of homologous miRNAs, either encoded by a sequence within the βSG gene (miRNA699q), or silenced at the post-transcriptional level (miRNA699a) in βSG^−/−^ cMabs. These miRNAs synergically control skeletal myogenesis. Whereas restoring wild-type (WT) βSG had only partial effects, knock-in of miRNA669a was adequate to prevent skeletal differentiation of cMabs [12, 13]. In our previous work, aberrant differentiation in βSG^−/−^ cMabs was evaluated mostly in terms of protein expression patterns with only preliminary reference to functional aspects. The present work undertakes a detailed characterization of excitation–contraction coupling in myotubes formed by *in vitro* differentiation of murine βSG^−/−^ cMabs (βSG^−/−^ myotubes). Particular attention was devoted to the detection of features suggesting residual myocardial differentiation. To this aim, βSG^−/−^ myotubes were compared, under uniform experimental conditions, to myotubes formed by a skeletal muscle cell line (C2C12) and to neonatal cardiac myocytes (CMs). C2C12 cells were used as the skeletal muscle prototype for consistency with our previous work on molecular characterization of aberrant βSG^−/−^ cMabs differentiation [12].

## Materials and methods

### Cell isolation and culture

cMabs were isolated from WT and βSG^−/−^ mice as previously described [12, 14]. cMabs clones were obtained from ventricle (H4V), atrium (ATG5) and aorta (AoA4), the number amplification passages at which WT and βSG^−/−^ clones were studied was similar (20 ± 2). Because AoA4 cMabs (either WT or βSG^−/−^) failed to form myotubes or to show any other sign of differentiation, they were discarded. Functional experiments on cMabs-derived myotubes were performed mainly on the H4V clone, but similar results were obtained with the ATG5 clone. cMabs and C2C12 cells were amplified in presence of DMEM 20% foetal bovine serum BS and differentiated in 3.5-cm Petri dishes in presence of low horse serum (2% HS) for 5 days before the experiments [12]. Neonatal (2–5 days p.n.) CMs were isolated from normal murine hearts as previously described and studied within 1–2 days from dissociation [15].

### Cell-shortening measurement

Cells shortening (twitch) was measured by video edge detection (Crescent Electronics, Salt Lake City, UT, USA) during field stimulation (2 Hz). Cells were superfused with Tyrode's solution containing (in mM): 154 NaCl, 4 KCl, 2CaCl_2_, 1 MgCl_2_, 5.5 D-glucose, 5 HEPES titrated to pH 7.35 with NaOH. Ca^2+^-free Tyrode's solution was obtained by substituting Ca^2+^ with Mg^2+^ (3 mM) plus EGTA (1 mM).

### Electrophysiological techniques

βSG^−/−^ and C2C12 myotubes, and single CM were voltage- or current clamped in the whole-cell configuration (Axopatch 200-A, Molecular Devices, Sunnyvale, CA, USA). Pipette (intracellular) solution contained (mM): 110 K^+^-Aspartate, 23 KCl, 0.2 CaCl_2_ (Ca^2+^ free = 10^−7^ M), 3 MgCl_2_, 5 HEPES KOH, 0.5 EGTA KOH, 0.4 GTP-Na salt, 5 ATP-Na salt, 5 creatine phosphate Na salt, pH 7.3. In I_CaL_ measurements, intracellular K^+^ was replaced by Cs^+^. Whole-cell series resistance was 5.7 ± 0.3 MΩ and the voltage error caused by their incomplete compensation was 2.4 ± 0.5 mV in the worst case (I_CaL_ recordings); both variables were similar among experimental groups. For I_CaL_ measurements extracellular K^+^ was replaced by TEA-Cl.

Current signals were filtered at 2 kHz and digitized at 5 kHz (Axon Digidata 1200; Molecular Devices). Current density was calculated by normalization to membrane capacitance (C_m_). The voltage dependence of I_CaL_ steady-state activation (d_∞_) was estimated from the current–voltage relation of peak I_CaL_ (I_peak_) by assuming complete activation and negligible inactivation at this time-point:


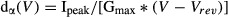


where G_max_ is the fully activated conductance, *V* is membrane potential and *V*_*rev*_ is the current reversal potential. d_∞_(*V*) data points were fitted by Boltzmann functions of the type:


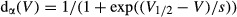


where *V*_1/2_ is mid-activation potential, and *s* is the slope factor (in mV). The time constants (τ) of I_CaL_ inactivation were estimated by mono-exponential fitting of I_CaL_ decay during the depolarizing step. Trace acquisition was controlled by a dedicated software (pClamp 8.0; Molecular Devices), analysis was performed by OriginPro 7 (OriginLab Corporation, Northampton, MA, USA). All recordings were made at 34 ±0.5°C. Voltage protocols are described in the figures.

### Ca^2+^ imaging experiments

Cells were incubated with 2 μM of the Ca^2+^-sensitive dye Fluo4-AM (Molecular Probes, Paisley, UK) for about 45 min. at room temperature and then resuspended in Tyrode's solution for about 15 min. Measurements were performed with a laser-scanning confocal microscope (Leica TCS SP2; Leica Microsystems, Wetzlar, Germany). Fluo4-AM was excited with an argon laser at λ = 488 nm, and the emitted fluorescence (F) was detected at λ > 512 nm. Changes in intracellular Ca^2+^ activity were expressed as changes in the F/F0 ratio (arbitrary units), where F0 is the mean field fluorescence recorded under baseline conditions. This set of experiments aimed to assess Ca^2+^ release from intracellular stores; therefore, all measurements were performed during superfusion with Ca^2+^-free Tyrode's solution.

The number of cells in which a given stimulus induced a [Ca]_i_ transient was measured from bidimensional (xy) images (sampling rate 1.2 Hz) from low magnification fields; the number of responsive cells was divided by the number of total cells in the field to obtain the percentage of stimulus responders as previously described [16]. The time course of [Ca]_i_ changes was obtained from the same images by monitoring fluorescence from individual cells in the field. Because of the slow kinetics of global Ca^2+^ release events, their time course could be reasonably defined even at this low sampling rate (Fig. 6).

Spontaneous unitary Ca^2+^ release events (Ca^2+^ sparks) were recorded at ×63 magnification in line-scan mode (sampling rate 0.8 kHz). Images were analysed by SparkMaster (ImageJ) software [17]. Automatic spark detection threshold (criteria) was slightly adjusted according to image quality, but was similar among experimental groups (3.56 ± 0.1, 3.5 ± 0.08 and 3.57 ± 0.11 for βSG^−/−^, C2C12 and CM respectively). The following spark parameters were measured: frequency (*N* of events/s*100 μm), amplitude (ΔF/F_0_), full width at half-maximal amplitude (μm, FWHM), full duration at half-maximal amplitude (ms, FDHM); time to peak (ttp) and decay time constant (τ, monoexp. fitting).

### Statistical analysis

Results are expressed as mean ± SEM; the significance of differences was tested by unpaired Student *t*-test for continuous variables and by the χ^2^ test for categorical ones. Sample size is reported in the figures; a value of *P* < 0.05 was considered significant.

## Results

### Characterization of undifferentiated cMabs

Molecular markers typical of mesoangioblasts [8] were equally expressed by WT (C3D10 clone) and βSG^−/−^ cMabs (Figure S1a). Early ‘cardiac’ markers (Myocardin and MEF2C) were expressed in cMabs but also in C2C12 cells (data not shown); therefore, they should not be considered as indicative of cardiac commitment.

Caffeine- or nicotine-induced whole-cell responses were totally absent in WT cMabs, occurred in a small percentage of βSG^−/−^ cMabs (caffeine 3.1%, nicotine 1.5%) and were even less frequent in C2C12 cells (caffeine 0.9%, nicotine 0.5%). This indicates the absence of a muscle-type Ca^2+^ store in the precursors at the pre-differentiation stage. On the other hand, ATP elicited Ca^2+^ transients were common even in undifferentiated βSG^−/−^ cMabs and C2C12 cells (96.6% and 86.3% respectively). Interestingly, 8–10 days of culture in differentiating medium were necessary before ATP-induced Ca^2+^ transients could be induced in WT cMabs (Figures S1c–S2b), to indicate the absence of even unspecific responses in undifferentiated WT cMabs.

### Characterization of differentiated cells/myotubes

After culture in differentiating medium for 5 days, both βSG^−/−^ cMabs and C2C12 cells fused to form multinucleated myotubes, as previously described [8].

Membrane capacitance was 110.5 ± 14 pF in WT cMabs (*n* = 13), 185.7 ± 18.8 pF in βSG^−/−^ myotubes (*n* = 23), 163.6 ± 12.8 pF in C2C12 myotubes (*n* = 27) and 44.1 ± 6.6 pF in CMs (*n* = 19). Even after 8–10 days of culture in differentiating medium, WT cMabs failed to form myotubes and remained as single non-contracting elements (Figure S1b). Preliminary analysis of their functional properties revealed inexcitability and poorly polarized membrane (resting *V*_m_ = −18.4 ± 2.2 mV, *n* = 13). Instantaneous and steady-state I/V relationships showed the absence of inward components (Figure S3a) and membrane current was unresponsive to nicotine (Figure S3b). Although oscillating spontaneously, cytosolic Ca^2+^ was unresponsive to caffeine (Figure S2a). These findings indicate that WT cMabs did not undergo differentiation; therefore, they were not subjected to the further functional evaluations aimed to define the differentiation fate of βSG^−/−^ cMabs as compared with that of C2C12 cells and CMs.

#### Ca^2+^ dependency of voltage-triggered contraction

**Fig. 1:**
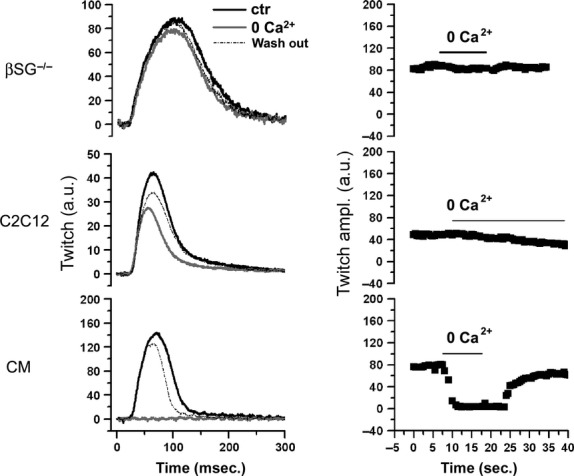
Dependence of contraction from extracellular Ca^2+^. *Left*: recordings of field-stimulated cell shortening (twitch, in arbitrary units) in βSG^−/−^, C2C12 myotubes and a CM during superfusion of Tyrode's solution containing 1.8 mM Ca^2+^ (Ctr, black line), 0 mM Ca^2+^+ 1 mM EGTA (grey line) and return (dot line). *Right*: time course of twitch amplitude in the three experimental conditions.

βSG^−/−^, C2C12 myotubes and individual CM contracted in response to electrical field stimulation (2 Hz) (Fig. 1). Extracellular Ca^2+^ is strictly required to couple sarcolemmal depolarization to force development in cardiac muscle (Ca^2+^-induced Ca^2+^ release, CICR), but not in the skeletal one (voltage-induced Ca^2+^ release, VICR). This set of experiments analysed Ca^2+^ dependency of voltage-triggered contraction in βSG^−/−^ myotubes and compared it with that of C2C12 myotubes and CMs.

After switching to Ca^2+^-free solution, stimulated cell shortening persisted almost unchanged for tens of seconds in βSG^−/−^ myotubes (Fig. 1 top) and decayed slowly in C2C12 ones (Fig. 1 middle). On the other hand, Ca^2+^ removal almost immediately abolished stimulated shortening in CMs (Fig. 1 bottom). In all cases, cell shortening at least partially recovered with Ca^2+^ readmission.

#### Presence of cholinergically induced contraction

Whereas cholinergic-receptor-operated channels (nicotinic receptors, nAChR) are highly expressed in skeletal muscle and mediate neurally induced contraction, cholinergic (muscarinic) receptors in cardiac muscle are exclusively metabotropic, with a modulatory role. Here, βSG^−/−^ myotubes are compared with C2C12 ones and with CMs in terms of contractile response to cholinergic stimulation.

**Fig. 2:**
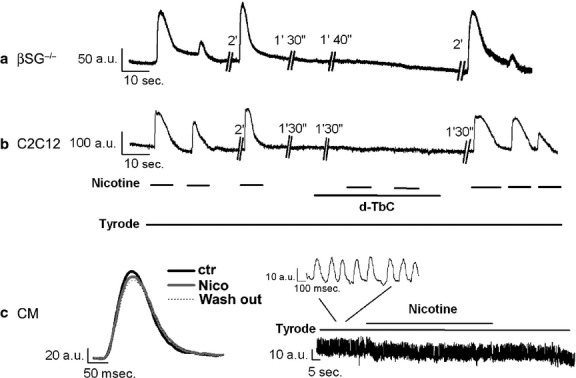
Contractile response to cholinergic stimulation. Cell length recordings in quiescent βSG^−/−^ (**a**) and C2C12 (**b**) myotubes. Nicotine (100 μM)-triggered cell contractions and their reversible suppression by the nicotinic receptor antagonist d-TbC (100 μM). (**c**) In CMs, nicotine neither triggered contraction during quiescence (*right*) nor changed the properties of contractions during electrical stimulation (*left*).

The cholinergic agonists nicotine (100 μM) and acethylcholine (100 μM, data not shown) consistently induced cell shortening in unstimulated βSG^−/−^ and C2C12 myotubes. Nicotine effect was blocked by the nAChR antagonist d-tubocurarine (100 μM d-TbC; Fig. 2a and b). Unstimulated CMs failed to respond to nicotine (data not shown); moreover, the latter had a negligible effect on either spontaneous or electrically triggered CMs shortening (Fig. 2c). In both βSG^−/−^ and C2C12 myotubes, nicotine repeatedly elicited full-fledged contractions only if applied at long intervals, as expected from nAChR desensitization [18].

Within βSG^−/−^ cMabs and C2C12 cultures, cells remaining as single elements (*i.e*. not forming myotubes) did not mechanically respond to nicotine challenge.

#### Effect of cholinergic stimulation on membrane current and voltage

In skeletal (but not cardiac) muscle, activation of nAChR induces a cationic transmembrane current, inward at resting membrane potentials and adequate to trigger action potentials. The effect of nicotine pulses (100 μM) on membrane current and potential was thus compared between βSG^−/−^ and C2C12 myotubes.

**Fig. 3:**
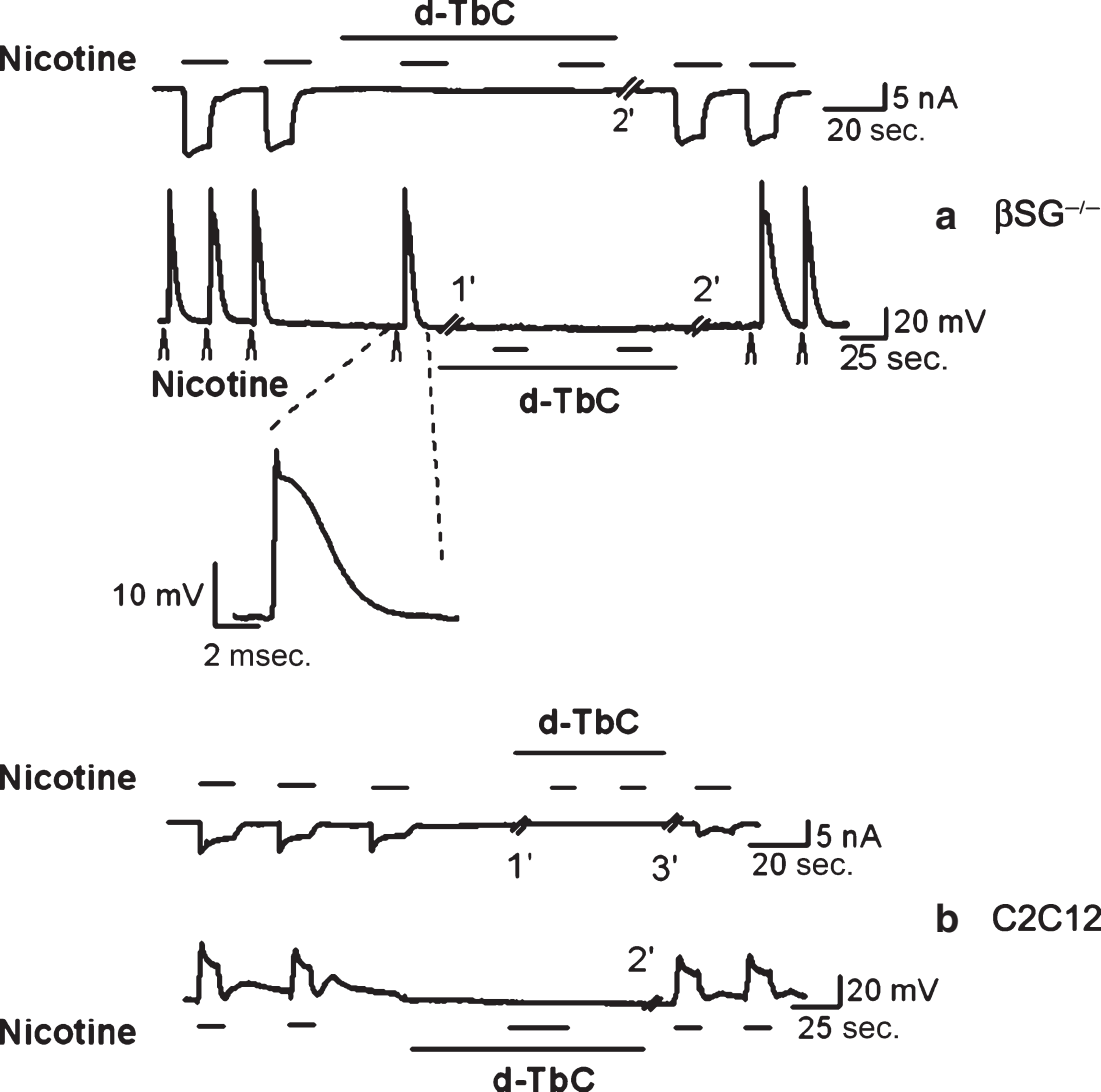
Cholinergic modulation of membrane current/voltage. Gap-free recordings in V-clamp (holding at −80 mV) and I-clamp (I = 0 pA) modes. In βSG^−/−^ (**a**) and C2C12 (**b**) myotubes, nicotine (100 μM) elicited inward current, which partially decayed during agonist application, and was reversibly abolished by d-TbC (100 μM). In I-clamp mode, nicotine elicited membrane depolarizations, also reversibly suppressed by d-TbC, which occasionally triggered full-fledged action potentials in βSG^−/−^ myotubes (a).

Total membrane current was recorded at a holding potential of −80 mV (Fig. 3). Nicotine challenge induced an inward current, partially decaying during sustained agonist exposure (Fig. 3a and b, upper records). Peak current density was comparable between βSG^−/−^ and C2C12 myotubes and values were 24.1 ± 4 pA/pF and 35.5 ± 7.3 pA/pF for βSG^−/−^ (*n* = 8) and C2C12 (*n* = 5) myotubes respectively (βSG^−/−^ *versus* C2C12, NS).

Under I-clamp conditions (I = 0) nicotine-induced membrane depolarization (Fig. 3 bottom records in each panel) and triggered action potentials in βSG^−/−^ and C2C12 myotubes (Fig. 3a, lower record). The effect of nicotine on membrane current and potential was reversibly blocked by *d*-tubocurarine in myotubes of both cell types (Fig. 3a and b).

#### Action potential analysis

**Fig. 4:**
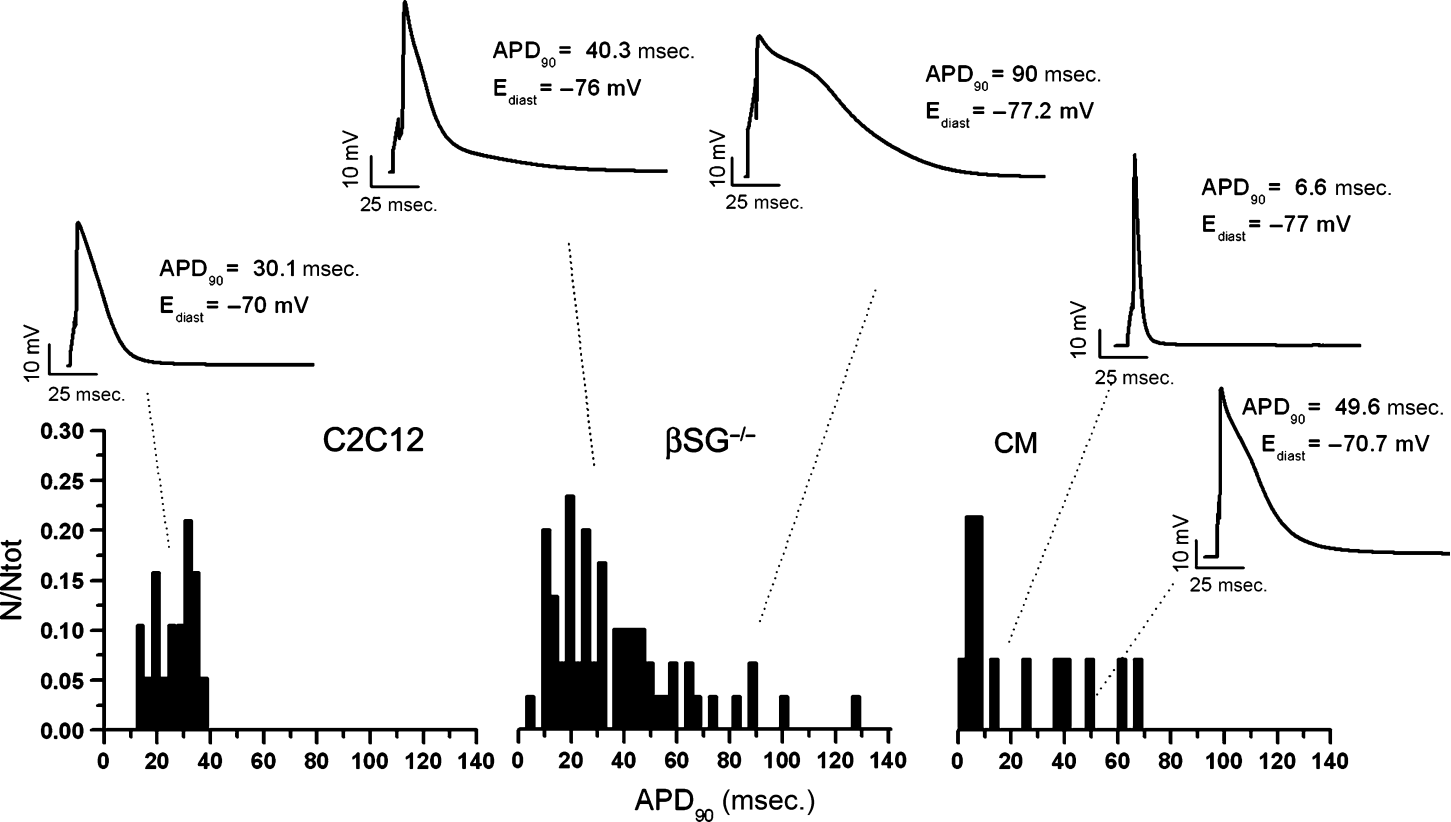
Action potential contour. *Top*: Representative examples of action potentials recorded during electrical stimulation in C2C12, βSG^−/−^ myotubes and CMs. *Bottom*: normalized distribution of action potentials durations in the three cell populations. APD_90_ = action potential duration at 90% repolarization; E_diast_ = diastolic potential, N/Ntot = fraction of observations in each APD_90_ interval.

Maturation of both skeletal and cardiac murine action potentials (AP) is accompanied by hyperpolarization of diastolic potential and shortening of AP duration (APD). The plateau phase, the fingerprint of large mammal's cardiac AP, largely disappears in mature rodent's cardiomyocytes, but it can still be observed in immature ones. This set of experiments, illustrated in Figure 4, compares action potentials of βSG^−/−^ myotubes, C2C12 myotubes and CMs.

In APs of C2C12 myotubes, repolarization was consistently monotonic; APD_90_ values were narrowly distributed around a mean of 26 ± 2 msec. (*n* = 19). In βSG^−/−^ myotubes, APD_90_ was 46 ± 5 msec. (*n* = 30; *P* < 0.05 *versus* C2C12 and CMs); its distribution, wide and skewed, also included very high values. The latter corresponded to APs with biphasic repolarization, *i.e*. displaying a more or less defined ‘plateau’ phase. In CMs, a majority of APs with fast and monotonic repolarization coexisted with a smaller proportion of longer APs with a discernible plateau phase; mean CMs APD_90_ was 29 ± 5 msec. (*n* = 19; NS *versus* C2C12).

Diastolic potential (E_diast_) was most variable in C2C12 myotubes and tended to be more positive (−63.7 ± 7.6 mV), than in either βSG^−/−^ myotubes (−70.6 ± 1.7 mV) or CMs (−71.1 ± 1.5 mV). Because of the scattered values in C2C12 myotubes, analysis of variance did not detect differences among the three cell types. No correlation was found between APD_90_ and E_diast_ values within each cell type.

#### Ca^2+^ current analysis

Faster and more complete inactivation of voltage-activated Ca^2+^ current is a distinctive feature of cardiac muscle as compared with skeletal one. Therefore, comparison of I_CaL_ properties may help in defining the functional phenotype of βSG^−/−^ myotubes.

I_CaL_ activation at various potentials was preceded by a 150 msec. step to −50 mV to inactivate I_CaT_ (holding potential = −90 mV). Activating steps of either 0.5 or 1 sec. in duration were used to accommodate the different I_CaL_ kinetics. Membrane capacitance (C_m_) was 180.2 ± 35.5 pF in C2C12 myotubes (*n* = 9), 117.4 ± 22 pF in βSG^−/−^ myotubes (*n* = 6) and 48.7 ± 10.4 pF in CMs (*n* = 9). I/V curves were obtained by plotting peak current density (I_m_/C_m_) *versus* activating potential.

**Fig. 5:**
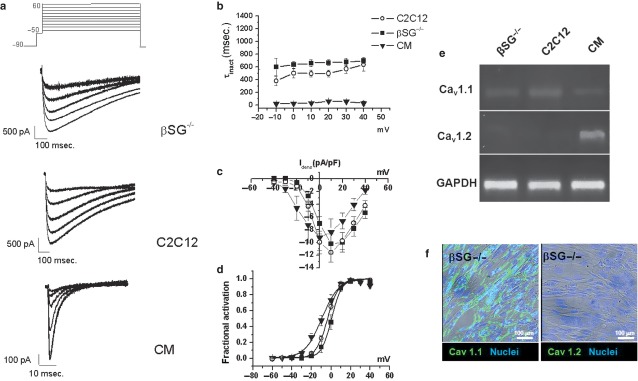
L-type Ca^2+^ current (I_CaL_) analysis and channel expression. (**a**) Representative I_CaL_ recordings in βSG^−/−^, C2C12 myotubes and CMs (voltage protocol in top panel); (**b**) V dependency of I_CaL_ inactivation time constants (τ_inact_); (**c**) Peak I_CaL_ I/V curves (top) and (**d**) steady-state activation curves (Boltzman fitting). (**e**) Expression (RT-PCR) of skeletal (Ca_v_1.1) and cardiac (Ca_v_ 1.2) Ca^2+^ channel isoforms (GAPDH as reference). Open circles: C2C12 (*N* = 9); filled squares: βSG^−/−^ (*N* = 6); filled triangles: CMs (*N* = 5). (**f**) Confocal immunofluorescence analysis of Cav 1.1 (skeletal) and cardiac Cav 1.2 (cardiac) isoforms in βSG^−/−^ myotubes. Signals for Ca_v_ (green) and nuclei (blue-Hoechst) are superimposed on the light transmission image.

As already evident by gross examination of sample recordings (Fig. 5a), inactivation kinetics was similar in βSG^−/−^ and C2C12 myotubes and differed from that of CMs (also notice the difference in the timescale). Figure 5b shows that inactivation time constants (τ_inact_) had a shallow dependency on membrane potential in all cell types, but were distinctly shorter in CMs (*n* = 9) than in βSG^−/−^ or C2C12 myotubes (*P* < 0.05 at all potentials, *n* = 7 and *n* = 12 respectively).

Peak current I/V relationship (Fig. 5c) was also similar between βSG^−/−^ and C2C12 myotubes, but was shifted towards negative potentials in CMs. Such a shift was quantified by Boltzman fitting of ‘activation curves’. In βSG^−/−^ myotubes (*n* = 6) *V*_1/2_ was similar to that of C2C12 myotubes (*n* = 9, −3.4 ± 2.2 mV *versus* −3.5 ± 1.1 mV, NS), but 6.3 ± 3.1 mV more positive than that of CMs (−9.7 ± 2.2 mV *P* < 0.05; *n* = 5); the slope factor was similar between βSG^−/−^ and C2C12 myotubes, but different for CMs (βSG^−/−^ 4.6 ± 0.8 mV; C2C12 5.5 ± 0.2 mV; CM 8 ± 0.4 *P* < 0.05). Maximal I_CaL_ density was slightly smaller in CMs (−8.3 ± 2.0 pA/pF) than in either βSG^−/−^ (−10.2 ± 1.6 pA/pF, *P* < 0.05) or C2C12 myotubes (−11.6 ± −1.5 pA/pF, *P* < 0.05).

Expression of skeletal (Ca_v_ 1.1) *versus* cardiac (Ca_v_ 1.2) Ca^2+^ channel mRNAs was studied by PCR. The Ca_v_ 1.1 transcript was equally expressed in C2C12 and βSG^−/−^ myotubes, and at a much lower level in CMs. The Ca_v_ 1.2 transcript was detected in CMs only (Fig. 5e). Immunolabelling of βSG^−/−^ myotubes revealed extensive expression of Ca_v_ 1.1. No Ca_v_ 1.2 signal could be detected in the same cell batch (Fig. 5f).

#### Function of the Ca^2+^ store

Sarcoplasmic reticulum (SR) function as a Ca^2+^ store was compared between βSG^−/−^ and C2C12 myotubes. This was achieved by evaluating whole-cell Ca^2+^ transients, elicited either by direct modulation of RyR (caffeine) and IP_3_R channels (IP3 increased by ATP), or by membrane depolarization (induced by nicotine or high [K^+^]_o_). Furthermore, the properties of Ca^2+^ sparks occurring in electrically quiescent cells (spontaneous releases) were studied in the three cell types. To focus on SR-dependent Ca^2+^ release, all measurements were carried out in the absence of extracellular Ca^2+^ (Ca^2+^-free solution).

**Fig. 6:**
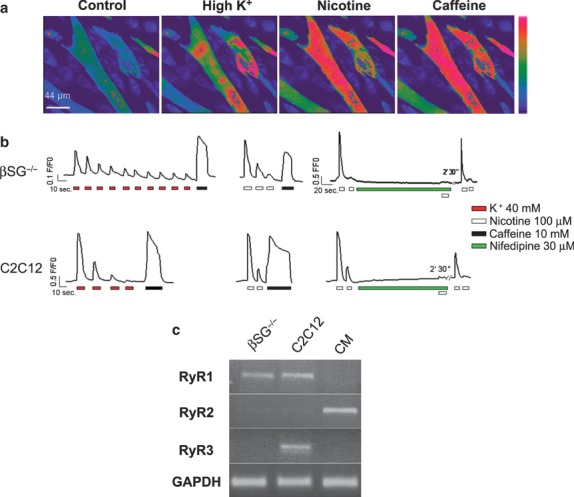
Intracellular Ca^2+^ transients. (**a**) confocal Ca^2+^ image ([Ca^2+^] colour scale *on the right*) of a βSG^−/−^ myotube field during exposure to Ca^2+^-free Tyrode and after addition of 40 mM extracellular K^+^ (High K^+^), 100 μM nicotine or 10 mM caffeine. (**b**) Ca^2+^ transients elicited by repeated challenge with high K^+^ (red bars), nicotine (white bars) or nicotine plus nifedipine (green bar) in βSG^−/−^ and C2C12 myotubes. The larger transient at the end of each record was induced by a caffeine pulse (bar) to measure SR Ca^2+^ content; (**c**) RyR isoforms expression patterns (RT-PCR) in βSG^−/−^, C2C12 myotubes and CMs (GAPDH as reference).

Caffeine and nicotine consistently induced Ca^2+^ responses in βSG^−/−^ and C2C12 myotubes (Fig. 6a). Therefore, the presence of Ca^2+^ responses triggered by nAChR stimulation, and mediated by RyR channels, can be related to cell differentiation for both precursor types.

In both βSG^−/−^ and C2C12 myotubes, repeated applications of either high [K^+^]_o_ or nicotine-triggered whole-cell Ca^2+^ transients of exponentially decreasing amplitude, as expected from depletion of the Ca^2+^ store. Nevertheless, within each cell type, the decay was faster with nicotine. Neither high [K^+^]_o_ nor nicotine-induced Ca^2+^ transients in the presence of 30 μM nifedipine (Fig. 6b), indicating the involvement of Ca_v_ channels. Even shortly after high [K^+^]_o_ pulses adequate to deplete the store, caffeine still elicited a large Ca^2+^ transient. This pattern has been reported for C2C12 myotubes [19] and interpreted to suggest that the Ca^2+^ compartment released by caffeine is at least not entirely accessible to VICR.

The decay of Ca^2+^ transients amplitude upon repeated high [K^+^]_o_ exposure was more complete and distinctly faster in C2C12 than in βSG^−/−^ myotubes, as quantified by the average number of exposures required for steady-state depletion (2.5 ± 0.4, *n* = 8 *versus* 9.4 ± 0.2, *n* = 12; Fig. 6b).

**Fig. 7:**
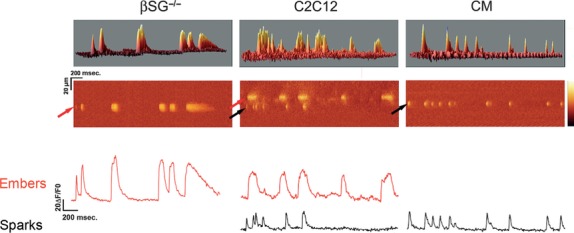
Elementary Ca^2+^ release events. Examples of elementary Ca^2+^ release events recorded from quiescent βSG^−/−^, C2C12 myotubes and CM; Top: 3D representation of line-scan images. *Middle*: original-line-scan images in colour scale (*right*); *Bottom:* [Ca^2+^] time course of the events shown in the middle panel (embers and sparks, see text for definitions).

Representative examples of Ca^2+^ sparks recordings in the three cell types are shown in Figure 7. The upper panel shows a 3D plot, in which normalized fluorescence intensity (amplitude) was digitized and represented on the vertical axis. Lower panels show raw-line-scan images (intensity represented by colour scale), aligned with the respective intensity profiles at the active release sites. Visual analysis of these examples reveals events with distinctly different durations; whereas short events prevailed in CMs and long ones in βSG^−/−^ myotubes, C2C12 myotubes were characterized by the presence of both short and long events, appearing at distinct release sites.

In previous work on skeletal muscle, short and long events have been classified as ‘sparks’ and ‘embers’, respectively, based on a FDHM cut-off of 50 msec. [20]. According to this criterion, embers were significantly represented in βSG^−/−^ and C2C12 myotubes (58% and 53.4% of total events respectively) and infrequent in CMs (21.5% of total events, *P* < 0.05 *versus* βSG^−/−^), where FDHM never exceed 60 msec. In βSG^−/−^ myotubes the same release site generated both sparks and embers; this was at variance with C2C12 myotubes, where each site generated a single class of events (*i.e*. spark or ember). Parameters means for all release events are compared between the three cell types in Table 1. Except for frequency, highest in βSG^−/−^ myotubes, all the parameters sharply distinguished CMs events from those of βSG^−/−^ and C2C12 myotubes, which were similar to each other. In the average, CMs events had larger amplitude, faster kinetics and smaller width as compared with those of the other cell types. Further characterization of elementary Ca^2+^ release events between the three cell types may be provided by comparison of distributions of spark image parameters, provided in the Supplement (Figures S4 and S5). In summary, means and distribution of spark image parameters converge in indicating gross similarity of elementary Ca^2+^ releases between βSG^−/−^ and C2C12 myotubes and a clear-cut difference between them and CMs; subtler differences were also observed between βSG^−/−^ and C2C12 myotubes.

**Table 1:** Properties of elementary Ca^2+^ release events

	βSG^−/−^ (*n* = 257)	C2C12 (*n* = 208)	CM (*n* = 254)
Amplitude (ΔF/F_0_)	0.47 ± 0.02	0.49 ± 0.01	1.07 ± 0.03*
FWHM (μm)	3.7 ± 0.1	3.8 ± 0.08	1.8 ± 0.04*
FDHM (ms)	94.5 ± 5.7	92.8 ± 8.7	41.7 ± 1.2*
τ (ms)	125.7 ± 14	120.4 ± 19.3	41.8 ± 1.5*
TTP (ms)	47.3 ± 3.1	55.9 ± 4.2	18.3 ± 0.8*
Frequency (N/s*100 μm)	3.67 ± 0.44	1.6 ± 0.4*	1.05 ± 0.3*

* *P* < 0.05 *versus* βSG^−/−^.

Expression of RyR isoforms was studied by PCR (Fig. 6c). Whereas C2C12 myotubes expressed both RyR1 and RyR3 mRNAs, βSG^−/−^ myotubes contained exclusively RyR1 transcript. RyR2, the only isoform found in CMs, was absent from the other cell types.

### Phenotype rescue by miRNA699a transduction

To test whether the WT phenotype could be rescued by re-expression of the sequence encoding for miRNA699a, βSG^−/−^ cMabs were transfected with lentiviral vector containing the sequence (LV-miRNA699a, Figure S6). Rescued βSG^−/−^ cMabs (R-βSG^−/−^ cMabs) were subjected to the same differentiation protocol and studied at the same times as non-transfected ones. R-βSG^−/−^ cMabs failed to form myotubes retaining a morphology similar to the wild-type cMabs. Ca^2+^ responses were evoked by ATP, but these cells were insensitive to either caffeine or nicotine (Figure S1c).

## Discussion

The findings of this study indicate that many functional properties assimilate βSG^−/−^ myotubes to C2C12 ones and, at the same time, distinguish them from CMs. These properties included: (1) dependency of voltage-triggered contraction on extracellular Ca^2+^; (2) presence of cholinergically induced contraction and Ca^2+^ responses; (3) membrane current and potential responses to cholinergic stimulation compatible with a robust expression of receptor-operated channels of the nicotinic type (blocked by d-tubocurarine); (4) I_CaL_ kinetics and activation voltage dependency; (5) features of elementary Ca^2+^ release events, both in terms of average parameters and of their distribution patterns (of particular notice, the presence of embers); (6) expression of Ca_v_ 1.1 and RyR1 transcripts, with virtual absence of Ca_v_ 1.2 and RyR2 ones.

βSG^−/−^ myotubes displayed many functional properties distinctive of skeletal muscle and, under homogeneous experimental conditions, shared them with myotubes formed by a line of skeletal muscle precursors (C2C12 cells). This observation is consistent with a robust commitment of the βSG^−/−^ mutant to the skeletal phenotype, as revealed by our previous molecular characterization [12]. Such a commitment was totally absent in WT cMabs and in βSG^−/−^ cMabs after rescue (by miRNA699a transduction); thus, it can be considered specific of the mutant genotype. At variance with previous studies [9, 21], we were unable to differentiate WT cMabs into cardiac myocytes. This observation is consistent with the evidence that transduction of βSG^−/−^ cMabs with miRNA669a, aimed to rescue the effects of βSG deletion, prevented skeletal differentiation but, at least *in vitro*, it failed to reinstate cardiac one [12]. Thus, our conclusions must be limited to indicate that the βSG gene may encode a suppressor of skeletal muscle differentiation, whose activity is mediated by miRNA669a. Considering the uniformity of conditions (cMabs source, culture conditions, *etc*.) inconsistency of cardiac differentiation of WT cMabs between the present and previous studies [12, 21] has no obvious explanation. In particular, because βSG^−/−^ and WT cMabs were studied after the same number of passages (see methods), cell ageing cannot be responsible for the discrepancy.

Even if sharing features typical of skeletal muscle, βSG^−/−^ and C2C12 myotubes were found to differ under several, more subtle, aspects. As compared with C2C12 myotubes, βSG^−/−^ ones had (1) larger variability in repolarization, presence of AP subpopulations with a discernible plateau phase; (2) higher frequency of elementary Ca^2+^ release events, which included more than one amplitude class and occurred as sparks and embers within the same release site; (3) absence of RyR3 expression; (4) slower depletion of Ca^2+^ store by repeated excitations.

Whereas the presence of a plateau phase is distinctive of adult cardiac muscle in larger mammals, in murine cardiomyocytes biphasic repolarization is limited to the foetal/neonatal stage and might indicate incomplete expression of I_to_, a repolarizing current appearing late during myocyte maturation [15, 22]. Nevertheless, as repolarization of C2C12 myotubes was consistently monotonic, the observation of biphasic repolarization in a subset of βSG^−/−^ myotubes might point to persistence of a cardiac trait in an otherwise skeletal phenotype. As I_CaL_ is the main current supporting the AP plateau, biphasic repolarization might depend on partial persistence of its cardiac isoform. However, in βSG^−/−^ myotubes I_CaL_ kinetics and the Ca^2+^ channel isoform expression were typically ‘skeletal’. Moreover, Ca_v_1.2 channel expression, obvious in CMs, could not be detected in βSG^−/−^ (Fig. 5e and f).

A higher frequency of elementary Ca^2+^ release events has been previously reported in skeletal myocytes from dystrophin knock-out mice and has been related to structural instability of the ‘junctional’ space, formed by the juxtaposition of sarcolemmal and SR membranes [23–25]. βSG belongs to the dystrophin complex and myocytes from βSG^−/−^ mice are mechanically weaker [12, 26]. Therefore, rather than reflecting a variation from the skeletal pattern, the abundance of spontaneous Ca^2+^ releases might be a direct consequence of βSG gene deletion on membrane mechanical stability.

The absence of RyR3 expression and slower depletion upon repeated excitations might be causally related. Indeed RyR3 channels are supposed to amplify VICR by adding a CICR component [27, 28]. Therefore, RyR3 expression might increase the fraction of SR Ca^2+^ content released by a single excitation. Interestingly, in mammalian skeletal muscle RyR3 are expressed in immature myocytes only, where they are thought to account for the larger incidence of spontaneous Ca^2+^ release events. [29]. Thus, at least according to the lack of RyR3 expression, βSG^−/−^ myotubes might appear as more mature than C2C12 ones. As opposed to RyR1, RyR3 is reported to mediate brief events [28, 30]; interestingly, release sites with brief events only were absent in βSG^−/−^ myotubes. Caffeine elicited large Ca^2+^ transients even when applied shortly after repeated depolarizations (by high K^+^ or nicotine) seemingly exhausting the releasable Ca^2+^ pool (Fig. 6b). This observation is consistent with previous reports in C2C12 myotubes, in which the finding was interpreted as the presence caffeine-releasable Ca^2+^ pool not accessible to VICR operation [19]. The occurrence of this phenomenon also in βSG^−/−^ myotubes, which do not express RyR3, may suggest the presence in these cells of a RyR1 population uncoupled from membrane voltage sensing.

## Conclusions

The present findings lead to conclude that deletion of the βSG encoding gene, which includes the miRNA699a/q sequences, removes an inhibitory control on cMabs differentiation towards a skeletal functional phenotype. Rescue of deletion effects by transduction of miRNA699a suggests that the latter is involved in the inhibitory control. These findings provide a functional counterpart to the previously reported role of miRNA699a in preventing skeletal-type gene expression in cMabs [12]. Although previously reported commitment of wild-type cMabs to myocardial differentiation [8, 9, 12] could not be reproduced in this study, subtler aspects of βSG^−/−^ cMabs function might suggest persistence of such a commitment.

## Supporting information

**Data S1** Expanded materials and methods.
