# Supplementary material for: Altered functional differentiation of mesoangioblasts in a genetic myopathy
Source: J Cell Mol Med. 2013 Feb 7;17(3):419–28. doi: 10.1111/jcmm.12023 (PMC3823023; doi:10.1111/jcmm.12023)
Supplement: Supplementary file 1 [file jcmm0017-0419-SD1.pdf]

Altered functional differentiation of cardiac progenitor cells in a genetic myopathy

Claudia Altomare[1], Lucio Barile[2], Marcella Rocchetti[1], Luca Sala[1], Stefania Crippa[3], Maurilio Sampaolesi[3] and Antonio Zaza[1].

1 Department of Biotechnologies and Biosciences, University of Milano-Bicocca, piazza della Scienza 2, 20126 Milano, Italy. 2 Cell Therapy Unit Cardiocentro Ticino, via Tesserete 48, 6900 Lugano, Switzerland. 3 Translational Cardiomyology, Stem Cell Research Institute, Catholic University of Leuven, Herestraat 49 B-3000 Leuven, Belgium.

Corresponding Author:
Antonio Zaza, M.D., F.E.S.C.
Dipartimento di Biotecnologie e Bioscienze, Università degli Studi Milano-Bicocca,
P.zza della Scienza 2, 20126 Milano, Italy
phone: +39 02 64483307; fax: +39 02 64483565
antonio.zaza@unimib.it

EXPANDED MATERIALS AND METHODS

Experimental interventions and substances

Ca^{2+} release through RyR channels was tested by challenge with 10 mM caffeine; cholinergic receptor-operated channels were activated by 100 μM nicotine and blocked by 100 μM *d*-tubocurarine. Exposure to high K^+ (40 mM) Tyrode's was used to induce receptor-independent membrane depolarization. Activation of voltage-dependent Ca^{2+} channels was prevented by 30 μM nifedipine. All substances were applied through an electronically timed, fast perfusion system.

Concentrated stock solutions were prepared by dissolving nicotine and *d*-tubocurarine in distilled water and nifedipine in ethanol. Aliquotes of stock solutions were added to Tyrode's solution to obtain final drug concentrations as indicated in results and figures (vehicle ≤0.3% of final volume). All substances were purchased from Sigma-Aldrich (St. Louis, MO).

Transcript analysis

mRNA was extracted from fresh isolated neonatal cardiomyocytes and from $\beta SG^{-/-}$ cMABS or C2C12 cell line 5 days after cells were plated under differentiation condition.

Trizol reagent (Invitrogen) was used as lysing buffer. mRNA samples were used as templates in random-hexamers-primed reverse transcription using the M-MuLV Reverse Transcriptase (Fermentas) with the following protocol: 10 min at 25°C followed by 60 min at 37°C and 10 min at 70°C.

PCR reactions were performed using 1μg cDNA from the RT step, 5 μL 10X PCR-buffer, 1 μL dNTP (25 μmol/L), 4 μL $MgCl_2$ (25 mmol/L), 2 μL of each primer pair and 1 μL DreamTaq polymerase (Fermentas) in a total volume of 50 μL. Primers sequences were designed as follow:

RYR-1:5'-GCTTAGCTGAGGTCTGCAGCTGG-3',5' AGGGGGTGTAGCACAGGATTTAT-3';

RYR-2:5'-GAATTCATCATGGATACTATACC-3',5'-TCATGCACATTATCTTCTGCAT-3';

RYR-3':5'-CCTGAGTTCACGACAAGCTACAG-3',5'-TAGCTGCTTAAAGCTTTTCAAGC-3';

Ca_v1.1:5'-GTTACATGAGCTGGATCACACAG-3',5'-ATGAGCATTTCGATGGTGAAG;

Ca_v1.2: 5'-CATCACCAACTTCGACAACTTC-3', 5'-CAGGTAGCCTTTGAGATCTTCTTC-3';

GAPDH: 5'-ACCACAGTCCATGCCATCAC-3'; 5'-TCCACCACCCTGTTGCTGTA-3'.)

The cycling conditions were 1 minute at 95°C followed by 30-35 cycles for 1 minute 94°C, 1 minute 58°C, and 1 minute 72°C in a PE 9600 PCR machine (Perkin Elmer, Waltham, MA). PCR products were separated on 2% agarose gel. PCRs were performed at various cycles to ensure linear amplification (data not shown), and minus-RT controls were performed to ensure specific amplification.

SUPPLEMENTARY RESULTS

Characterization of undifferentiated cMabs:

For description see manuscript page 8

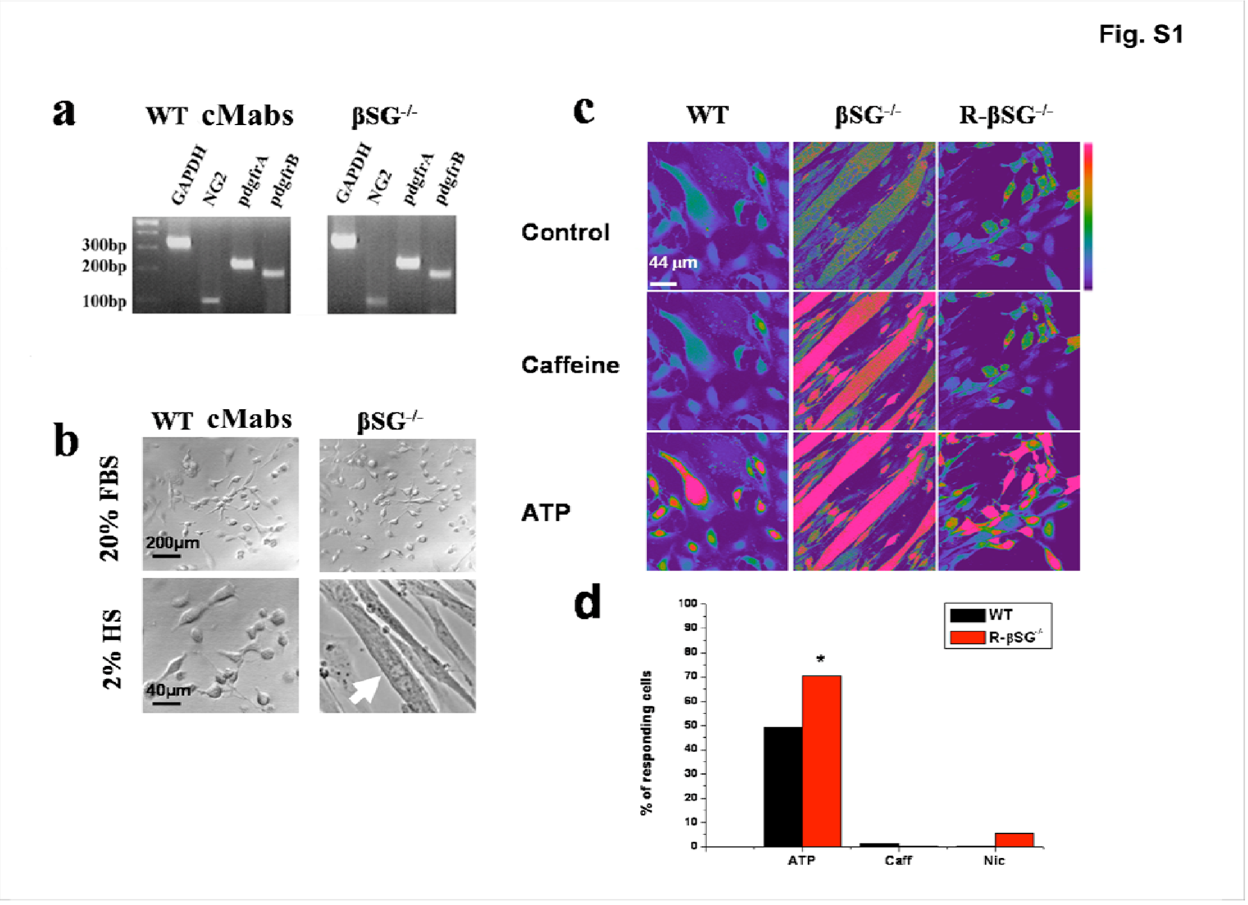

Fig. S1 Comparisons between WT, βSG$^{-/-}$ and rescued (R-βSG$^{-/-}$) cMabs: a) pre-differentiation RT-PCR analysis of pericyte molecular markers (NG2, pdgfA and pdgfB) supporting the common origin of WT and βSG$^{-/-}$ from cMabs. b) light transmission images of WT and βSG$^{-/-}$ cMabs cultured for 7 days in the presence of serum-complemented (20%FBS) and serum-free (2% HS) media. Myotube formation (arrow) in serum-free conditions is visible for βSG$^{-/-}$ cMabs only. c) Examples of caffeine- and ATP-induced Ca^{2+} responses (color scale at right) after exposure to differentiating conditions (2%HS). Confocal images were recorded during Ca^{2+}-free superfusion. Whereas WT and R-βSG$^{-/-}$ appear as individual cells, βSG$^{-/-}$ form myotubes. d) Comparison between WT and R-βSG$^{-/-}$ in terms of prevalence of Ca^{2+} responses to ATP, caffeine and nicotine (from the experiments in c); * $p<0.05$ WT *vs* R-βSG$^{-/-}$

Fig. S2

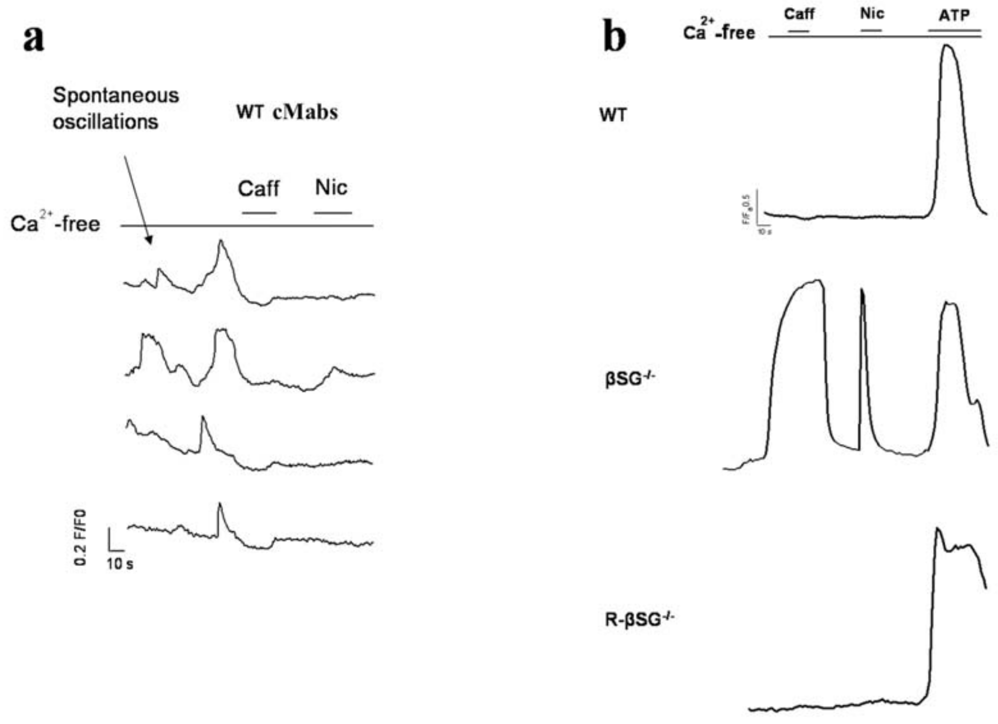

Fig. S2 Examples of Ca^{2+} signals in WT, $\beta SG^{-/-}$ and R-$\beta SG^{-/-}$ cells

a) while spontaneous Ca^{2+} oscillations occurred in WT cMabs (15 days in 2%HS) responses to caffeine and nicotine were virtually absent; b) while in $\beta SG^{-/-}$ myotubes Ca^{2+} release was induced by caffeine, nicotine and ATP, only ATP-induced Ca^{2+} release occurred in WT and R-$\beta SG^{-/-}$ cells

Fig. S3

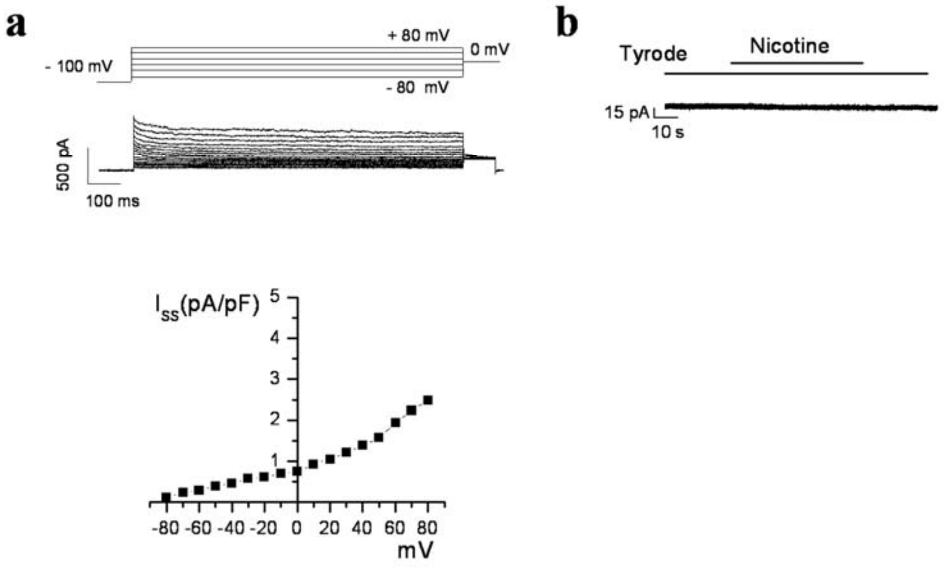

Fig. S3 Membrane currents in WT cMabs

Patch-clamp recordings of total membrane current (I_m) in WT cMabs: a) example of I_m recordings (V protocol in the inset) and the respective I/V relationship. b) nicotine failed to induce I_m changes (holding potential -80 mV)

Distributions of spark image parameters

Distributions of spark image parameters for $\beta SG^{-/-}$, C2C12 myotubes and CM are shown in Fig. S4.

Fig. S4

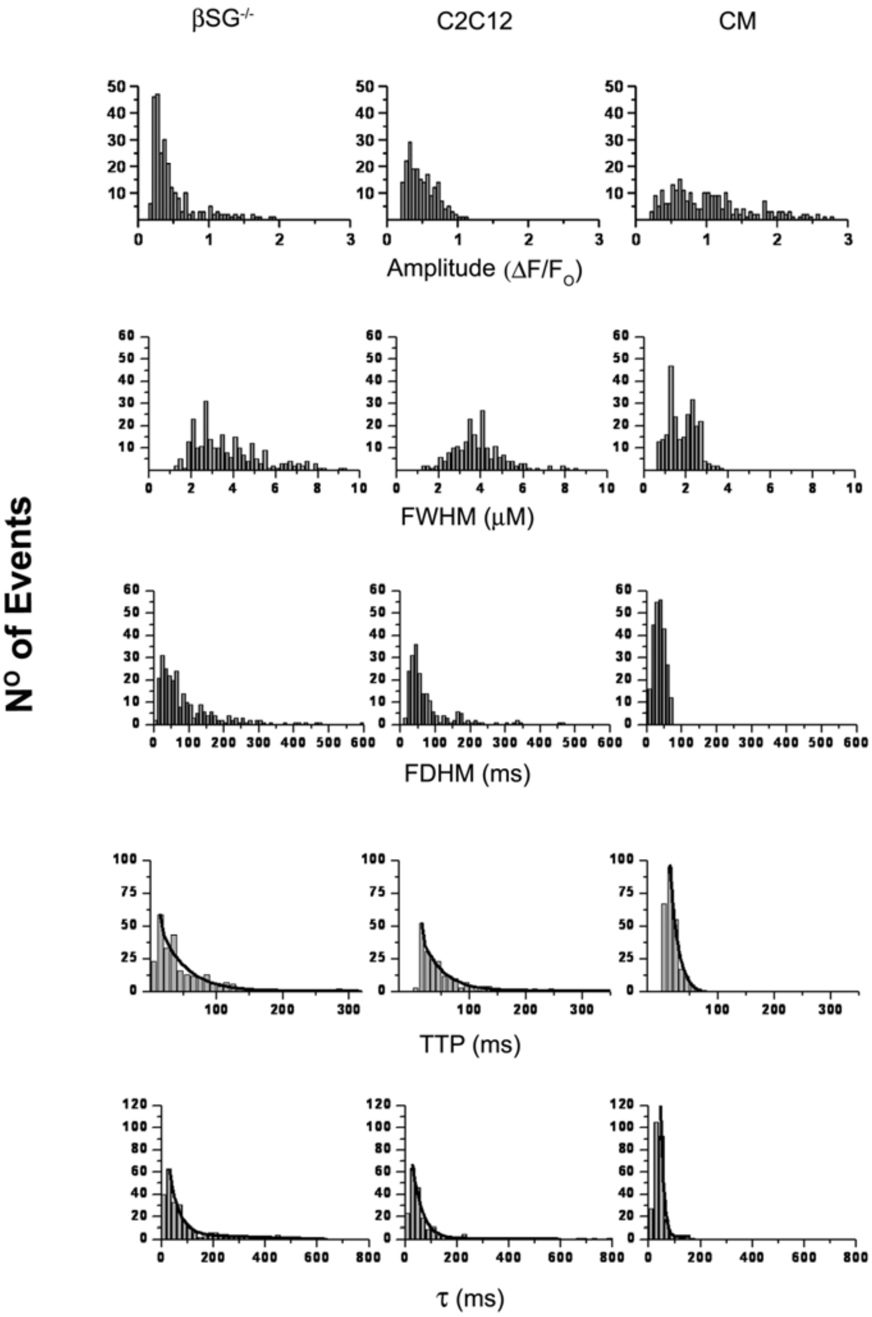

Fig. S4 Parameter distributions for elementary Ca^{2+} release events: Distributions of the parameters characterizing Ca^{2+} release events in the three cell types. *From top*: amplitude ($\Delta F/F_0$), full width at half-amplitude (FWHM, μm), full duration at half-amplitude (FDHM,

ms), time to peak (TTP, ms) and time constant of decay (τ, ms). Distributions of TTP and τ were fitted with mono- or bi-exponential functions (solid lines) to identify different classes of events (sparks *vs* embers).

Visual analysis of spark image distribution parameters reveals common aspects in $\beta SG^{-/-}$ and C2C12 myotubes, which differentiate them from CMs. For instance, spark amplitude distribution, non-modal in $\beta SG^{-/-}$ and C2C12 myotubes, is clearly modal in CMs; moreover, distributions of time parameters (FDHM, TTP and τ), although non-modal in all cases, have in CMs a smaller variance.

Distribution of spark image parameters may not be representative of the actual properties of unitary Ca^{2+} release events because of indetermination in the position of release sites (in the y and z dimensions) with respect to the scan line [1,2]. Indeed, stereotyped sparks (i.e with invariant parameters) may generate image parameter distributions with different shapes (non-modal, modal etc.) according to the imaging bias. However, theory predicts that, if sparks have constant properties (stereotyped), their image amplitude ($\Delta F/F_0$) should be inversely related to width (FWHM) [1]. Such relationship was instead direct in the case of $\beta SG^{-/-}$ ($R = 0.43$, $m = 0.09$; $p<0.05$) and completely absent in C2C12 myotubes and in CMs ($R<0.01$ and NS in both cases) (Fig S5). In the case of $\beta SG^{-/-}$ myotubes, regression residuals identified two distinct spark image populations, one of them likely generated by larger sparks, as indicated by the strong and positive relation between image amplitude and FWHM (regress. coeff. for $\beta SG^{-/-}$ 1 = 0.3 and for $\beta SG^{-/-}$ 2 = 0.04; $p<0.05$) (Fig S5).

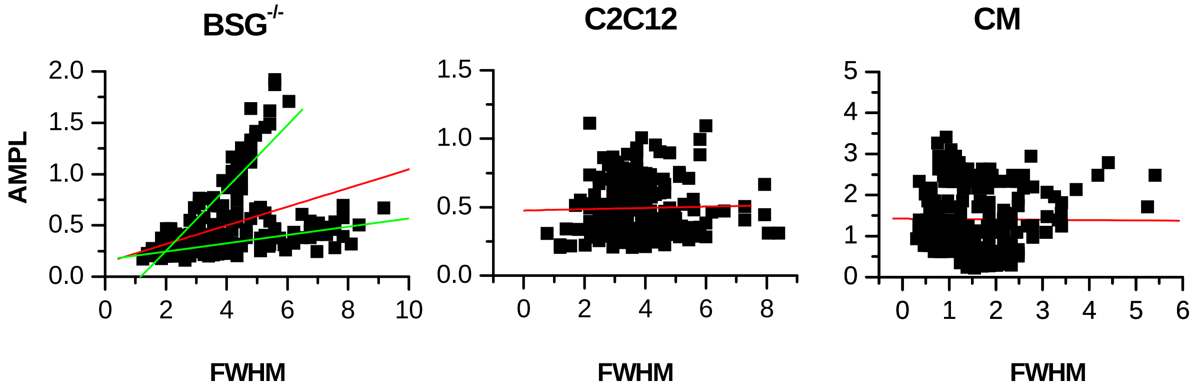

	R	m	p	βSG$^{-/-}$ 1		βSG$^{-/-}$ 2	
				R	m	R	m
βSG$^{-/-}$	0.43	0.09	<0.05	0.82	0.3	0.73	0.04
C2C12	0.029	--	NS				
CM	0.008	--	NS				

Fig. S5. Correlation between spark amplitude and FWHM. Regression analysis of Amplitude *vs* FWHM in βSG$^{-/-}$, C2C12 myotubes and CMs. In all panels the red lines show global data fitting. βSG$^{-/-}$ data were further divided (βSG$^{-/-}$1, βSG$^{-/-}$2) according to the sign of global fitting residuals; the green lines in the βSG$^{-/-}$ panel show fitting of each subgroup separately. Correlation and regression coefficients are reported in the table. R = correlation coefficient; m = regression coefficient.

Therefore, although spark distribution properties should be interpreted with caution because of imaging bias, the differences in the distributions shown in Fig S4 may reflect actual differences in properties of Ca^{2+} release units between cell types.

Fig. S6

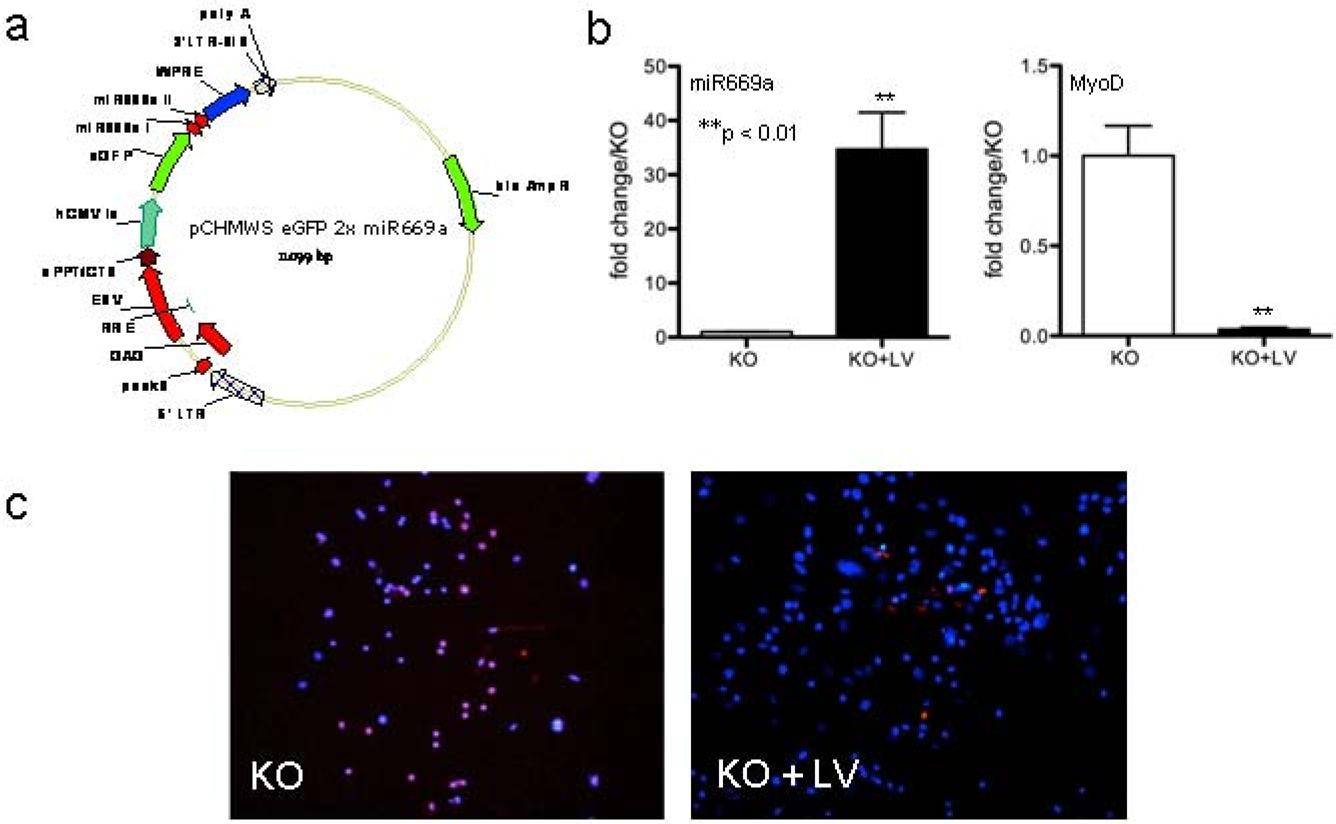

Fig. S6 A Schematic representation of lentiviral vector LV-CMV-eGFP-miR669a2x used to overexpress miR669a in *Sgcb*-null clones. **B**. Taqman analysis for miR669a expression in *Sgcb*-null cells transduced with LV-CMV-eGFP-miR669a2x (KO+LV) compared to untransduced *Sgcb*-null cells (KO). miR16 was used for sample normalization (N=3) (left panel); qPCR analysis for MyoD expression in *Sgcb*-null cells transduced with LV-CMV-eGFP-miR669a2x (KO+LV) compared to untransduced *Sgcb*-null cells (KO). GAPDH was used for sample normalization (N=3) (right panel). **C**. Immunoflurescence analysis for the expression of MyoD (in red) in *Sgcb*-null cells transduced with LV-CMV-eGFP-miR669a2x (KO+LV) compared to untransduced *Sgcb*-null cells (KO). Nuclei are counterstained with Hoechst (in blue).

Reference List

1. **Cheng H, Song LS, Shirokova N et al.** Amplitude distribution of calcium sparks in confocal images: theory and studies with an automatic detection method. *Biophys J*. 1999; 76: 606-17.
2. **Pratusevich VR & Balke CW.** Factors shaping the confocal image of the calcium spark in cardiac muscle cells. *Biophys J*. 1996; 71: 2942-57.
